# The utility of automated volume analysis of renal stones before and after shockwave lithotripsy treatment

**DOI:** 10.1007/s00240-020-01212-8

**Published:** 2020-09-14

**Authors:** Helen Wei Cui, Tze Khiang Tan, Frederikke Eichner Christiansen, Palle Jörn Sloth Osther, Benjamin William Turney

**Affiliations:** 1 Oxford Stone Group, University of Oxford, Oxford, UK; 2 Ninewells Hospital and Medical School, University of Dundee, Dundee, DD1 9SY UK; 3 Department of Urology, Urological Research Center, Lillebaelt Hospital, University of Southern Denmark, Vejle, Denmark

**Keywords:** Clinical insignificant residual fragments, Extracorporeal shockwave lithotripsy, Renal stone, Stone volume

## Abstract

This study aimed to evaluate the additional utility of an automated method of estimating volume for stones being treated with shockwave lithotripsy (SWL) using computed tomography (CT) images compared to manual measurement. Utility was assessed as the ability to accurately measure stone burden before and after SWL treatment, and whether stone volume is a better predictor of SWL outcome than stone diameter. 72 patients treated with SWL for a renal stone with available CT scans before and after treatment were included. Stone axes measurement and volume estimation using ellipsoid equations were compared to volume estimation using software using CT textural analysis (CTTA) of stone images. There was strong correlation (*r* > 0.8) between manual and CTTA estimated stone volume. CTTA measured stone volume showed the highest predictive value (*r*^2^ = 0.217) for successful SWL outcome on binary logistic regression analysis. Three cases that were originally classified as ‘stone-free with clinically insignificant residual fragments’ based on manual axis measurements actually had a larger stone volume based on CTTA estimation than the smallest fragments remaining for cases with an outcome of ‘not stone-free’. This study suggests objective measurement of total stone volume could improve estimation of stone burden before and after treatment. Current definitions of stone-free status based on manual measurements of residual fragment sizes are not accurate and may underestimate remaining stone burden after treatment. Future studies reporting on the efficacy of different stone treatments should consider using objective stone volume measurements based on CT image analysis as an outcome measure of stone-free state.

## Introduction

Current evidence and treatment guidelines for the management of renal tract stones, including the American Urological Association (AUA), European Urology Association (EAU) and United Kingdom NICE recommendations, are guided by the size of the stone both at diagnosis, and of the remaining fragments after initial treatment. However, there is no agreed standardised method of defining stone size both in terms of how stone axis lengths or stone volumes should be measured, and which imaging modality should be used to visualise stone size [1–4].

Previous studies investigating the efficacy of different treatment modalities for renal tract stones have shown large heterogeneity in the methods used to define outcomes from treatment, including choice of imaging modality, method of measuring stone burden and the size definition of ‘clinically insignificant residual fragments’ or CIRFs [5, 6]. The recent evidence review for the NICE guidelines show the difficulties of comparing outcomes between different stone treatment modalities when the definition of ‘stone-free’ can vary between studies [7]. Most studies do include the presence of CIRFs as a ‘stone-free’ outcome after treatment, and recommendations for choosing extracorporeal shockwave lithotripsy (SWL), retrograde intrarenal surgery (RIRS) or percutaneous nephrolithotomy (PCNL) are based on these stone-free rates. However, there is no agreement on whether all CIRFs are significant and therefore only completely ‘stone-free’ should be included as a successful outcome of treatment, or whether some residual fragments may be more significant than others based on risk of future symptoms and need for retreatment [8].

Stone volume is now considered to be a more accurate and reliable method of measuring stone burden, which is also becoming more feasible with the increased use of computed tomography of the kidneys ureter and bladder (CT KUB) and ultra-low-dose CT KUB [9]. However, both the use of 3D reconstruction, and the manual calculation of stone volume using axes measurements, are not routinely performed [10]. Single axis measurement of stone burden is still the norm in clinical practice, and forms the basis of studies and guidelines for renal stone management [11, 12]. Little is known on how measuring the volume of residual stones may impact on our understanding of CIRFs.

This study compared measurement of stone burden on CT KUB images using a semi-automated software tool to estimate stone volume, versus traditional methods of estimating stone size using manual axis measurements, both before and after SWL treatment. The ability of stone volume to help predict for SWL outcome, and the significance of residual fragments based on stone axis versus volume measurement was also evaluated.

## Patients and methods

Retrospective data collection of consecutive patients who had undergone SWL at a single institution between 2010 and 2014 were screened. Inclusion criteria were adult patients with renal calculi treated with SWL who had a CT KUB performed before and after SWL treatment. This is same cohort used in previous study by Christiansen et al. [13].

### Manual measurements of stone size

CT KUB DICOM files were analysed using MicroDICOMviewing software. The measure tool was used to manually measure stone axis lengths. The axial slice that subjectively had the largest area was chosen, and the maximum diameter in the *x* and *y* perpendicular axes, and *x* axis on coronal view were used. Manual calculation of stone volume was estimated from the following three equations for a volume of an ellipsoid: the scalene ellipsoid formula (*π*/6 × *a* × *b* × *c*), the oblate ellipsoid formula (*π*/6 × *a* × *a* × *c*) and the prolate ellipsoid formula (*π*/6 × *a* × *b* × *b*), where *a* is the equatorial diameter, *b* is the polar diameter, and *c* is the third measurable diameter [10].

### Semi-automated measurements of stone size

**Fig. 1:**
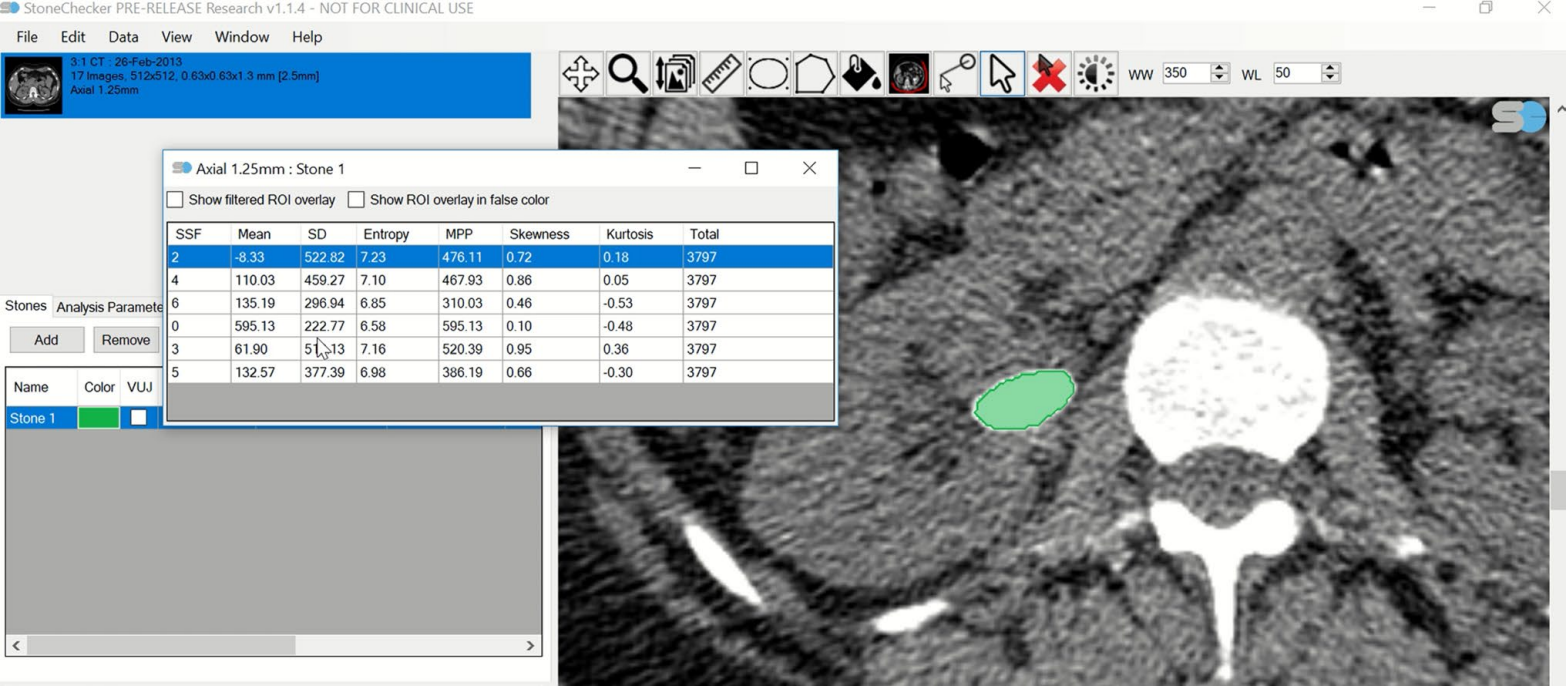
Example of the generation of computed tomography textural analysis variables using the StoneChecker Software. The green area represents the semi-automated region of interest generated by the software

DICOM files were also analysed using proprietary CT texture analysis (CTTA) software (StoneChecker Software Limited, Radstock, UK) [14]. This software semi-automatically populates the region of interest (ROI) pertaining to the stone on all axial images that include the stone. This is performed by setting a Hounsfield unit (HU) threshold that would discriminate for any pixels that could represent the stone rather than surrounding tissue or urine. The pixels contained in the ROI across all image slices containing the stone are used to calculate measures of stone burden including the major horizontal axis length, major vertical axis length, cross-sectional area of the largest slice, the volume of each stone and the total number of pixels present (Fig. 1). The volume is estimated by the software by counting all the available pixels in all the ROIs drawn for the stone and multiplying this by the size of the pixel and slice thickness as obtained from the DICOM metadata.

### SWL treatment

SWL treatment was performed by an experienced urologist or uroradiologist using the Medical Modulith® SLX F2 (Storz Medical AG, Switzerland) lithotriptor. During the procedure, fluoroscopy and/or ultrasonography was used for stone targeting using the ‘extended focus’ setting. The energy of the shock waves started at 1.0 J and slowly increased to the maximum energy level of 6.0 J depending on the stone. The number of shock waves delivered ranged from 800 to 4000 depending on the response seen on imaging at the time of treatment. Most patients received 2000–3000 shock waves at a frequency of 1.0–1.5 Hz.

### Outcome of SWL

Outcome of SWL, as defined by the clinical team was based on post-treatment CT KUB imaging and measurement of any remaining fragments seen using either manual measurement or CTTA software. ‘Stone-free’ was defined as no stones remaining after SWL on follow-up CT KUB imaging. CIRFs were defined by the host institution as fragments ≤ 4 mm. There was no prescribed way of measuring size of residual fragments by the host institution [10].

### Statistical analysis

The strength of the relationship between the manual versus the semi-automated stone burden measurements was assessed using Pearson r correlation. Binary logistic regression was performed for the degree of variance in the SWL treatment outcome explained by different measurements of stone burden. All analyses were performed using IBM SPSS Statistics for Windows, version 24.

## Results

### Patient characteristics

72 patients undergoing SWL were included in this study for analysis as outlined in Table 1. 69/72 (96%) of SWL treatments were for 1 stone in the same location. The median major axis length was 6.92 mm (IQR = 5.7–8.9), and the median volume was 113 mm^3^ (IQR = 62–276). Most of the stones were located in the lower pole (45.8%) and renal pelvis (40.3%). Most patients (83.3%) required only one session of SWL. The success rate of SWL defined as ‘completely stone-free’ on CT KUB follow-up imaging was 44.4%. Follow-up CT KUB to determine outcome of SWL was performed at a median of 61 days (IQR = 42–76) after the last SWL treatment. 11/72 (15%) of cases required additional treatment which included 10 cases requiring RIRS and one case requiring PCNL.

**Table 1:** Demographic and clinical outcome

	Total	Outcome		
		Completely stone-free	Stone-free with CIRFs	Not stone-free
No. treated cases	72	32 (44)	37 (51)	35 (49)
No. male (%)	42/72 (58.3)	20/32 (62.5)	25/37 (67.6)	18/35 (51.4)
No. left side (%)	34/72 (47.2)	15/32 (46.9)	19/37 (51.4)	16/35 (45.7)
No. stone location (%)				
Upper pole	2/72 (2.8)	2/32 (6.3)	2/37 (5.4)	
Midpole	7/72 (9.7)	4/32 (12.5)	4/37 (10.8)	5/34 (14.7)
Lower pole	33/72 (45.8)	18/32 (56.3)	21/37 (56.8)	15/35 (42.9)
Renal pelvis	29/72 (40.3)	8/32 (25.0)	10/37 (27.0)	14/35 (40.0)
Missing	1/72 (1.4)			1/35 (2.9)
Measures of stone burden				
No. of stones				
1	69/72 (96)	32/32 (100)	36/37 (97.3)	33/35 (94.3)
2	3/72 (4)		1/37 (2.7)	2/35 (5.7)
Stone axis length				
Major axis length; mm^a^	6.9 (5.7–8.9 [3.4–20.1])	6.2 (5.2–8.1 [3.4–11.8])	6.3 (5.2–8.1 [3.4–13.8])	7.7 (6.0–10.8 [3.9–20.1])
Vertical axis length; mm^a^	7.5 (5.7–10.5 [2.6–21.0])	6.7 (5.3–8.1 [3.7–12.7])	6.8 (5.0–8.6 [3.7–12.7])	9.0 (6.0–11.4 [2.6–21.0])
Stone volume(s); mm^3a^	113 (62–276 [21–1820])	74 (56–141 [21–350])	80 (58–168 [21–518])	199 (77–396 [8–1820])
Total no. of pixels^a^	220 (139–485 [30–3224])	190 (131–391 [65–966])	200 (142–446 [65–1615])	238 (133–511 [30–3224])
No. SWL sessions				
1	60/72 (83.3)	30/32 (93.8)	35/37 (94.6)	27/35 (77.1)
2	10/72 (13.9)	1/32 (3.1)	1/37 (2.7)	7/35 (20.0)
3	1/72 (1.4)			
≥ 4	1/72 (1.4)	1/32 (3.1)	1/37 (2.7)	1/35 (2.9)

CIRFs, clinically insignificant residual fragments; CT KUB, computed tomography kidneys ureter bladder; SWL, shockwave lithotripsy

^a^Values were measured using the StoneChecker software. *‘*Stone-free with CIRFs’ are patients who are completely stone-free or patients who had CIRFs after treatment. ‘Completely stone-free’ is a subset of ‘Stone-free with CIRFs’ for patients who are completely stone-free only. ‘Not stone-free’ are patients who have residual fragments after treatment which are larger than CIRFs. ‘Total’ includes the sum of patients who are ‘Stone-free with CIRFs’ and ‘Not stone-free’

### Manual vs. semi-automated measurements of stone burden on CT KUB

Comparison of semi-automated volume estimation using the StoneChecker software versus the manual estimations of stone volume showed that all stone size and volume variables (across the three equations used for ellipsoid volume calculation) showed strong positive correlation between these two methods (Table 2), with a Pearson correlation coefficient of *r* > 0.8. More specifically, manual stone volume calculation based on the oblate and scalene formulas for an ellipsoid showed correlation with software estimated volume of *r* > 0.9.

**Table 2:** Correlation between a manual versus semi-automated method of measuring stone size

Variables	Manual method	Semi-automated method	Pearson *r* correlation
Major horizontal axis length (mm)^a^	6.6 (5.4–9.0 [3.0–20.8])	6.9 (5.7–8.9 [3.4–20.1])	0.97
Major vertical axis length (mm)^a^	7.2 (4.7–9.3 [2.6–16.3])	7.5 (5.7–10.5 [2.6–21.0])	0.88
Manual volume estimation using different equations for the volume of an ellipsoid			
Oblate	161 (71–379 [13–3347])	113 (62–276 [21–1820])	0.94
Scalene	100 (40–247 [10–1420])		0.94
Prolate	65 (24–181 [1–1172])		0.83

Values are median (IQR [range])

^a^Values were measured from the largest cross-sectional slice of the stone manually. The manual method of calculating stone volume used the following formulas for an ellipsoid: oblate ellipsoid (*π*/6 × *a* × *a* × *c*), scalene ellipsoid (*π*/6 × *a* × *b* × *c*) and prolate ellipsoid (*π*/6 × *a* × *b* × *b*)

### Classification of CIRFs usual manual vs. semi-automated size measurements

Outcome of SWL was classified in one of three categories: ‘completely stone-free’, ‘stone-free with CIRFs’ and ‘not stone-free’ based on the original classification using manual stone axis measurements of follow-up CT KUB images. For cases with an outcome of ‘stone-free with CIRFs’ and ‘not stone-free’, the total volume of any remaining fragments was estimated using the StoneChecker software. Based on volume estimation using StoneChecker, there was an overlap of stone volumes between the ‘not stone-free’ and ‘stone-free with CIRFs’ groups (Table 3). There were three stones that were originally classified as ‘stone-free with CIRFs’ which were subsequently found to have a total stone volume *larger* than the volume of the smallest fragments (8.34 mm^3^) found in the ‘not stone-free’ group. This showed that the original classification of treatment outcome as ‘stone-free with CIRFs’ may have underestimated the total remaining stone burden of some cases. Conversely, measurement of major axis length showed that three cases with an original outcome of ‘not stone-free’ had a major axis diameter of less than 4 mm which would therefore have qualified to have an outcome of ‘stone-free with CIRFs’.

**Table 3:** Major horizontal length and volume of the five smallest stone fragments remaining in the ‘not stone-free’ (a) and ‘stone-free with CIRFs’ groups (b) measured using the StoneChecker software. Results in bold in section (b) show that 3 cases classified as ‘stone-free with CIRFs’ had a larger volume than the smallest volume in the ‘not stone-free group’

(a) Post-SWL remaining stone size and volume in the ‘not stone-free’ group	
Major horizontal length (mm)	Volume (mm^3^)
2.387	8.34
2.535	10.61
3.516	10.78
4.238	16.18
4.386	19.11

(b) Post-SWL remaining stone size and volume in the ‘stone-free with CIRFs’ group	
Major horizontal length (mm)	Volume (mm^3^)
1.642	2.38
2.304	7.05
3.516	**10.30**
3.672	**11.73**
3.854	**26.07**

CIRF, clinical insignificant residual fragment; SWL, extracorporeal shock wave lithotripsy

### Volume vs axis length for predicting stone-free outcome

Traditionally, the major horizontal axis length serves as the predictive parameter for the outcome of SWL. As can be expected, the cases in this study which showed an outcome of ‘completely stone-free’, overall, had a smaller major horizontal axis length than those cases with a treatment outcome of ‘not stone-free’. Analysis of the stone size variables for the outcome of SWL using binary logistic regression demonstrated a significant contribution from StoneChecker measured variables of stone volume, major axis length, vertical axis length, slice area; as well as significant contributions from manually measured variables of minor axis length and stone volume (Table 4). However, the CTTA measurements of stone volume had the highest statistical predictive value as compared to the rest of the parameters (Nagelkerke *R* square = 0.217). This indicates that stone volume variable can explain 21.7% of the variance in the outcome of stone-free or not. In comparison, major axis length as measured using StoneChecker showed the ability to explain 9.9% of the variance in the outcome of stone-free or not.

**Table 4:** Comparison of the prediction ability for an outcome of ‘stone-free’ after extracorporeal shock wave lithotripsy using the volume and stone axis measurements generated by StoneChecker

Variables	Sig	Exp (*B*)	95% CI for Exp (*B*)		Nagelkerke *R* Square
			Lower	Upper	
Major axis length (mm)	0.036	0.806	0.66	0.986	0.099
Vertical axis length (mm)	0.034	0.828	0.696	0.986	0.100
Area of largest slice/ROI (mm^2^)	0.014	0.964	0.936	0.993	0.156
Stone volume (mm^3^)	0.008	0.994	0.99	0.999	0.217

The higher value of Nagelkerke *R* Square indicates better prediction of stone-free rate outcome

CI, confidence interval; ROI, region of interest

## Discussion

In this study, measurement of stone volume using an objective, semi-automated CTTA software programme provides more information on stone burden before and after SWL treatment. Before treatment, stone volume has more predictive ability for successful SWL outcome than axis measurements. After SWL, stone burden can be underestimated if based on axis measurements of fragments alone. The total volume of fragments may be more helpful to determine whether any residual fragments are significant and likely to need retreatment.

As CT becomes the standard imaging modality to diagnose renal tract stones, the utility of measuring stone volume using CT software is increasingly recognised as important for planning treatment and predicting treatment outcome. Previous studies have used several different methods for estimating stone volume using CT images including, 3D reconstruction of the stone to measure axis lengths and then applying an ellipsoid formula [9, 15]; using third party CT software to estimate volume [10, 16]; and using a HU thresholding and voxel counting technique [17]. Using stone volume instead of axial measurements may be a better predictor of treatment outcome, as small differences in manual axis measurements may lead to much larger volume changes [9, 16]. This may be more applicable with increasing size of stone, as found by Finch et al*.* [10]. As maximum stone diameter increases, stone volume estimation using ellipsoid volume equations become less accurate [10]. These results reflect our findings as shown in Table 2 of the ellipsoid equation volume estimation versus the semi-automated volume estimation. The manual volume estimates in our study vary significantly depending on which equation is used, although the relationship between manual and automated methods is strong across all equations [10]. The technique in this study also employs voxel counting as well as automated selection of the region of interest based on HU thresholding. This has advantages over other techniques which require manual drawing of the region of interest or manual measurement of axis lengths which may be inaccurate and are less reproducible [16].

Stone size has been shown in previous studies to be a strong predictor for SWL outcome amongst other stone and patient related factors [18–23]. In almost all studies, a measurement of stone axis diameter is used. Our study supports previous evidence that stone volume is a stronger predictor of SWL outcome than axis length [9]. On binary logistic regression, using StoneChecker measurements, stone volume explains slightly more of the variation in the SWL treatment outcome of stone-free or not than the major axis length (Nagelkerke *R* squared of 0.099 for major axis length compared to 0.217 for stone volume). Although estimated volume using the CTTA software did correlate highly with manually estimated volume (*r* > 0.9), the use of software volume calculation is likely to be less arduous than manual measurement of 3 stone axes lengths and, our analysis suggests the CTTA estimated volume is slightly more predictive for outcome than manually estimated volume. This technique may be especially useful for larger stones with irregular outlines, or where there is more than one stone, as the software can sum the volumes of all stones to be treated.

The utility of stone volume measurement to plan treatment is already recognised as important [24]. However, less has been studied on the volume measurement of residual fragments after treatment. In this study, three stones that were originally classified as ‘stone-free with CIRFs’ actually had a total stone volume larger than the volume of the smallest residual fragments in the ‘not stone-free group’. This shows that classifying stone outcome as ‘stone-free with CIRFs’ can lead to misclassification of some stones that are actually larger than those classified as ‘not stone-free’. Accurate stone volume measurement of residual fragments may have more value in defining outcome from stone treatment [17, 25]. This study showed an overlap of stone volumes between cases classified as ‘stone-free with CIRFs’ and ‘not stone-free’, i.e. had residual fragments after treatment that were > 4 mm and therefore not CIRFs. For example, one of the cases of ‘not stone-free’ had a residual fragment with a major horizontal length of 4.238 mm and stone volume of 16.18 mm^3^, whereas a case of ‘stone-free with CIRFs’ had a major horizontal length of 3.854 mm and a larger stone volume of 26.07 mm^3^.

Defining outcome of stone treatment based on ‘completely stone-free’ or ‘not stone-free has been advocated by some, to reduce confusion over the use of the term CIRFs [26]. However, studies of the natural history of CIRFs after SWL and RIRS have shown that that the majority of CIRFs (approx. 56–78%) do spontaneously pass or requires no further treatment [8, 26–29]. This still leaves a significant proportion (approx. 29–50%) which does require retreatment. A recent study followed 232 subjects after RIRS who had residual fragments of any size for 12 months with the availability of follow-up imaging. This found that 29% of subjects required intervention during the follow-up period and likelihood of reintervention was predictable based on fragment size [27]. Given that most cases do not require reintervention for CIRFs, removing this term entirely may not be helpful for clinical decision making on which patients may require a secondary intervention after initial SWL. It is likely there is close relationship between size of residual fragments and need for reintervention and therefore accurate measurement of residual fragment size is important. This can help to both compare treatment outcomes in studies of stone interventions and inform further research on predicting which residual fragments are likely to require further treatment.

The authors acknowledged that this study possesses limitations. Firstly, this study is retrospective in nature and patients could only be included who had a CT both before and after SWL treatment. This may have biased to include a population of patients who had a lower success rate from SWL as most straightforward cases of SWL do not usually require CT after treatment. The timing of the post-treatment CT was therefore not standardised, and the presence and size of residual stone fragments would be influenced by time passed after SWL treatment. However, this does not change the method by which remaining stone fragments after SWL were measured. Secondly, there was a lack of standardisation of the original method of measuring CIRFs by the clinical team which leads to exaggerated inaccuracies in stone burden estimation after treatment. Thirdly, the sample size in this study is small due to the data available from this single centre. It is difficult to draw conclusions on how stone size is estimated in other centres to understand the applicability of these results. However, of the stone studies in the literature, stone size measurement is often not reported in the methodology as a standardised method.

## Conclusion

This study shows the potential benefit of objective measurements of stone burden using a semi-automated approach both before, and after, SWL treatment. This method of measuring stone volume is less time consuming than manual methods and, offers a more standardised approach to comparing the efficacy of different stone treatments as part of clinical trials.

## Data Availability

Not applicable.

## Abbreviations

CIRF: Clinically insignificant residual fragments
CT KUB: Computed tomography of the kidney, ureters and bladder
CTTA: Computed tomography textural analysis
HU: Hounsfield unit
IQR: Interquartile range
PCNL: Percutaneous nephrolithotomy
RIRS: Retrograde intrarenal surgery
ROI: Region of interest
SWL: Extracorporeal shockwave lithotripsy
